# Clinical and Surgical Applications of Large Language Models: A Systematic Review

**DOI:** 10.3390/jcm13113041

**Published:** 2024-05-22

**Authors:** Sophia M. Pressman, Sahar Borna, Cesar A. Gomez-Cabello, Syed Ali Haider, Clifton R. Haider, Antonio Jorge Forte

**Affiliations:** 1 Division of Plastic Surgery, Mayo Clinic, Jacksonville, FL 32224, USA; 2 Department of Physiology and Biomedical Engineering, Mayo Clinic, Rochester, MN 55905, USA; 3 Center for Digital Health, Mayo Clinic, Rochester, MN 55905, USA

**Keywords:** artificial intelligence (AI), ChatGPT, diagnosis, management, deep learning, machine learning, surgical specialties

## Abstract

**Background:** Large language models (LLMs) represent a recent advancement in artificial intelligence with medical applications across various healthcare domains. The objective of this review is to highlight how LLMs can be utilized by clinicians and surgeons in their everyday practice. **Methods:** A systematic review was conducted following the Preferred Reporting Items for Systematic Reviews and Meta-Analyses guidelines. Six databases were searched to identify relevant articles. Eligibility criteria emphasized articles focused primarily on clinical and surgical applications of LLMs. **Results:** The literature search yielded 333 results, with 34 meeting eligibility criteria. All articles were from 2023. There were 14 original research articles, four letters, one interview, and 15 review articles. These articles covered a wide variety of medical specialties, including various surgical subspecialties. **Conclusions**: LLMs have the potential to enhance healthcare delivery. In clinical settings, LLMs can assist in diagnosis, treatment guidance, patient triage, physician knowledge augmentation, and administrative tasks. In surgical settings, LLMs can assist surgeons with documentation, surgical planning, and intraoperative guidance. However, addressing their limitations and concerns, particularly those related to accuracy and biases, is crucial. LLMs should be viewed as tools to complement, not replace, the expertise of healthcare professionals.

## 1. Introduction

Large language models (LLMs) are emerging as an innovative force in the field of artificial intelligence (AI) with the promise to reshape the landscape of healthcare. But what are LLMs? LLMs are AI systems that can understand and generate human-like text [1,2]. Analogous to the neural structure of the human brain, LLMs operate through intricate configurations of virtual neurons known as neural networks [2]. Among the prevalent architectural frameworks utilized within LLMs are transformers such as generative pre-trained transformers (GPTs) [3]. These frameworks facilitate the coherent composition of textual information into meaningful and contextually appropriate sentences. Furthermore, like a brain, LLMs possess the capability to adapt and learn from data. This learning process is integral to their development and effectiveness. Through iterative exposure to a training dataset, LLMs refine their predictive abilities by anticipating subsequent words in a sequence, evaluating their predictions against actual outcomes, and adjusting their parameters accordingly until they achieve a high level of proficiency [4,5].

**Figure 1:**
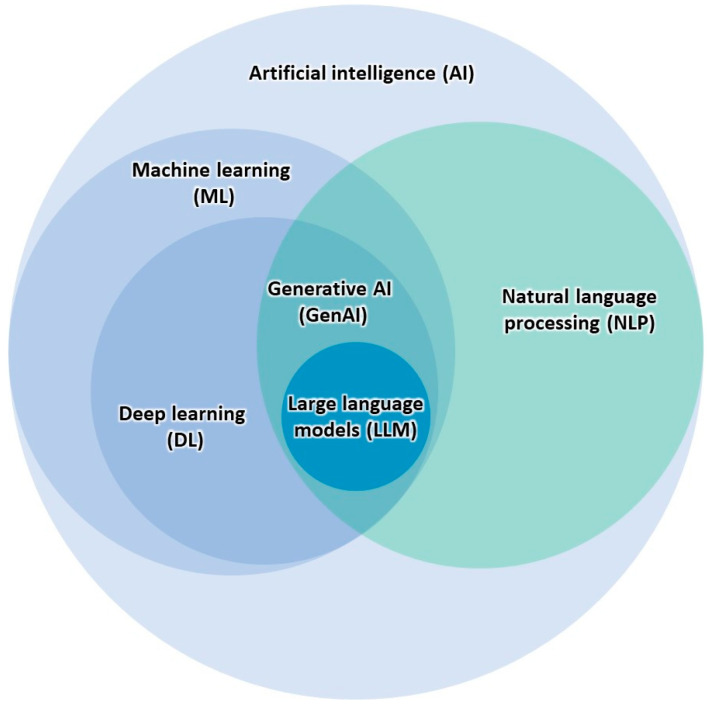
Relationships between AI technologies, including LLMs.

As pre-trained models, LLMs utilize natural language processing (NLP) and deep learning technology [4] (Figure 1). These models are lauded for their language comprehension and ability to efficiently convey information with a high degree of competence. With these comprehension capabilities, the exploration of these models and their applications in medicine has already begun [6], sparking cautious excitement in the healthcare industry [7]. This excitement has fueled discourse on how these models can impact healthcare and enhance patient outcomes [8]. After its public release in late 2022, OpenAI’s ChatGPT [9] (San Francisco, CA, USA) has quickly become one of the most well-known LLMs. ChatGPT acts as a knowledgeable conversation partner, comprehending inquiries, mimicking human-like understanding, and responding in a diverse range of communication styles [10]. ChatGPT’s successful passage of the United States Medical Licensing Examination (USMLE) [11] demonstrates its medical reasoning and contextualization abilities, underscoring its potential in the field of medicine. The current literature suggests that ChatGPT holds promise in a multitude of medical applications, including clinical diagnosis and treatment, medical education support, and public health guidance [12]. There have been multiple proposed benefits to using LLMs like ChatGPT, including optimized clinical decision-making, streamlined workflow, improved patient care, and enhanced communication between healthcare professionals [3]. Despite this growing area of interest, there are few studies that provide a comprehensive overview of clinical and surgical applications of LLMs. Previous reviews have explored AI in healthcare broadly [13,14,15,16] or within specific specialties or tasks [17,18,19,20], but do not typically focus on LLMs specifically. Conversely, some studies have exclusively explored ChatGPT applications, neglecting to discuss other LLMs. Although these studies provide significant contributions to the discussion of AI utility in medicine, there remain gaps in the literature.

As the use of LLMs becomes increasingly prevalent in healthcare, it is important to explore the full breadth of medical applications. This prompts the question: what are the healthcare applications of LLMs within clinical and surgical contexts? There is a need for a clinician- and surgeon-focused review to explore the extent, impact, and challenges associated with LLM implementation in these domains. The objective of this systematic review is to highlight how LLMs can be utilized by clinicians and surgeons in their everyday practice while shedding light on the practical limitations and ethical considerations. By doing so, this study aims to provide an overview of the potential applications and limitations that clinicians and surgeons are likely to encounter as healthcare moves into the digital age. By exploring these applications and filling in research gaps, this study endeavors to contribute to the ongoing discourse on this rapidly evolving field and provide insights to inform further research and practice.

## 2. Materials and Methods

### 2.1. Search Strategy

A search strategy to optimize the retrieval of relevant articles was employed. This study focused on publications that discussed the clinical and surgical applications of LLMs, such as ChatGPT, for clinicians and surgeons within human medicine. Appropriate keywords were combined using Boolean operators to develop the following search input: “((“large language model”) OR (“ChatGPT”) OR (“chat GPT”) OR (“generative AI”) OR (“generative artificial intelligence”)) AND ((diagnos*) OR (intervention) OR (management) OR (“clinical medicine”) OR (“medical decision making”) OR (((decision) AND (making)) AND ((clinical) OR (medical)))) AND ((surger*) OR (surgical))”.

### 2.2. Data Sources and Databases Searched

Six databases consisting of the Cumulative Index to Nursing and Allied Health Literature (CINAHL), Excerpta Medica Database (EMBASE), Google Scholar, PubMed, Scopus, and Web of Science were searched on 14 September 2023, with the same search string. To capture emerging trends and reflect the significant advancements of LLM architectures. like GPT’s, in the last few years, only articles published after 2018 were considered. As Google Scholar typically sorts by relevance, only the first 100 results were included in the identification process. All identified articles were imported into EndNote software (Version 20.4.1) for reference management.

### 2.3. Study Eligibility and Selection Process

Predetermined eligibility criteria guided the study selection process. Eligible studies were articles that explored the clinical or surgical applications of LLMs for physicians. Since this review focused specifically on applications for physicians, articles that focused primarily on LLM utilization for related healthcare fields (e.g., dentistry or nursing), research, medical education, and patient use were considered out of scope and therefore excluded. Similarly, articles that did not specifically focus on LLMs, such as articles exploring other AI technologies, were also excluded. As long as the record represented a peer-reviewed journal article, there were minimal restrictions regarding the study design or article type. Since this is a qualitative systematic review with the objective to identify and detail, rather than quantify, LLM applications, redundancy was not considered a major limitation that would prevent the inclusion of systematic reviews that may cite the included original studies. However, to minimize the inclusion of potential subjective opinion pieces, Letters to the Editor that did not include some original contribution or data were excluded. Additional reasons for exclusion included duplicate records, non-peer-reviewed articles, and non-English studies.

After the database search, identified references were compiled into a citation manager. Duplicate records were then removed. Subsequently, the screening of records based on title and abstract resulted in the initial removal of records deemed to be irrelevant as per the eligibility criteria. A subsequent eligibility assessment was performed to identify studies that met the inclusion criteria.

**Figure 2:**
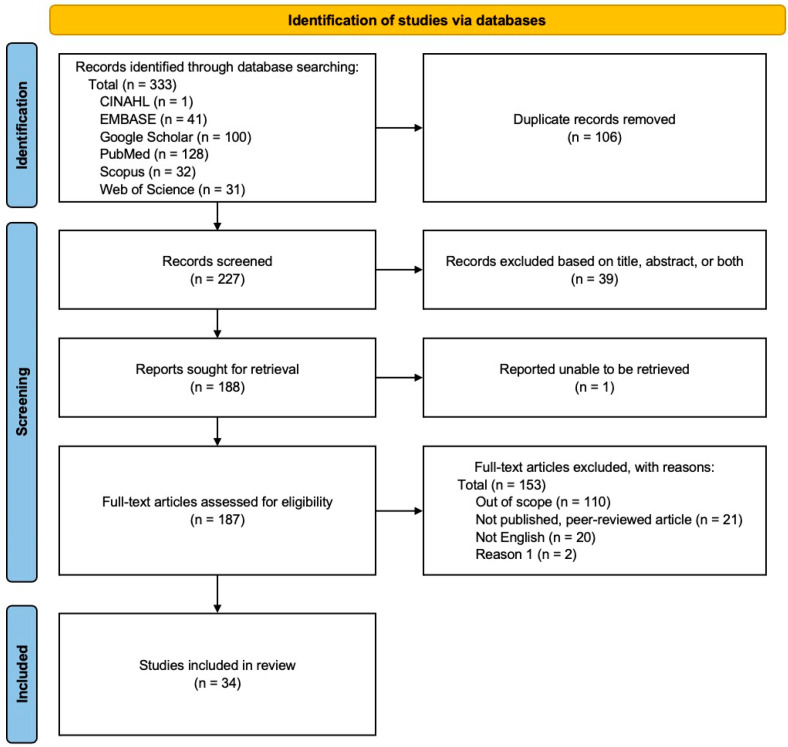
Modified 2020 PRISMA flow diagram outlining the article identification and eligibility assessment process for this systematic review.

No protocol for this systematic review was registered. However, this qualitative systematic literature review followed the organizational framework provided by the Preferred Reporting Items for Systematic Reviews and Meta-Analyses (PRISMA) guidelines [21] (Figure 2). The increased transparency and more structured, rigorous methodology of a systematic review is what drove the decision to adopt this approach over other review methods.

### 2.4. Data Collection and Synthesis

Study details were systematically extracted and then organized utilizing Microsoft Excel (Redmond, WA, USA). The following details were extracted: first author, specialty, study design, objective, main clinical applications of LLMs, main limitations of LLMs, and article conclusion. These data were then analyzed, summarized, and synthesized to offer a comprehensive overview.

## 3. Results

### Characteristics of Included Studies

The literature search yielded a total of 333 results, of which 34 met the eligibility criteria. Of the included studies, there are fourteen original research articles, seven systematic reviews, eight non-systematic reviews, four letters to the editor, and one interview. Although a quarter of articles were not specialty-specific, orthopedic surgery/spine surgery (*n* = 3), otolaryngology/head and neck surgery (*n* = 3), and plastic surgery (*n* = 3) were the most represented specialties within the included studies.

Commonly cited LLM applications included diagnosis, generating differential diagnoses, guiding treatment decisions and further workup, augmenting physician knowledge, and interpreting laboratory and imaging results. Thirty-one articles adequately discussed the limitations of LLM use, with concerns regarding the accuracy and quality of responses being one of the most commonly cited limitations. A summary of included studies is displayed in Table 1.

Confidence analysis, assessment of heterogeneity and risk of bias were not applicable due to the nature of the review and the types of included studies.

## 4. Discussion

### 4.1. Applications of LLMs in Clinical Settings

**Figure 3:**
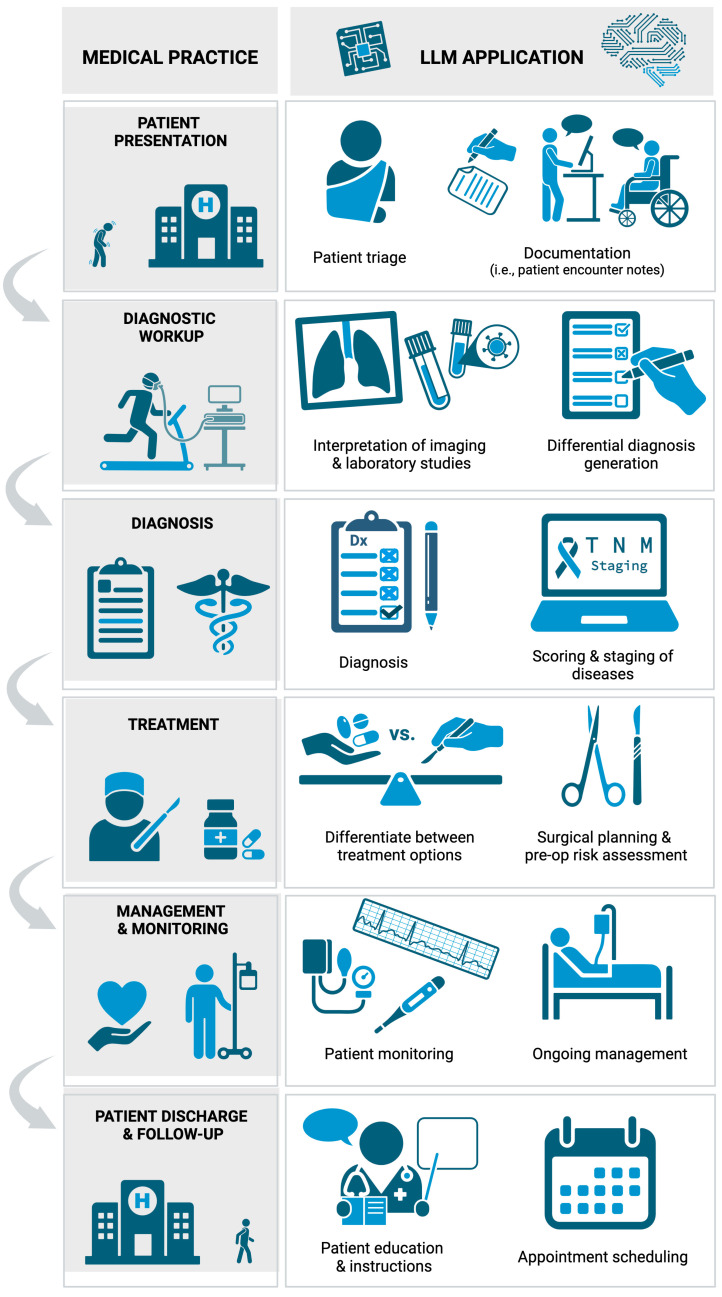
Applications of LLMs within clinical practice. Created with BioRender.com.

LLMs like ChatGPT demonstrate a wide variety of applications within clinical medicine (Figure 3). One of the most promising clinical applications is the ability to assist in the diagnostic process. LLMs have the capacity to comprehensively evaluate a broad range of clinical data, including symptoms [6,8], medical history [12,23], and diagnostic test results [35], which enables them to swiftly generate potential diagnoses. This can assist healthcare professionals in making informed decisions, accelerating the diagnostic process [3,6,23,47]. To provide a more precise diagnosis, LLMs can be effectively integrated with a range of medical scoring, staging, or grading systems [42]. They can contribute to tasks such as establishing TNM staging for cancer patients [34], and calculating metrics like the Glasgow Coma Scale (GCS) and other neuro-scores for stroke patients [25]. Diagnostic accuracy can be further strengthened by using LLMs to interpret laboratory tests [24] and radiologic studies [25,36]. Furthermore, multiple studies noted that clinicians could employ LLMs as a resource for patients to gain a better understanding of their test results [6,24,33,34,36,45].

These diagnostic capabilities extend across multiple specialties and pathologies. For example, Rajjoub et al. found that ChatGPT accurately addressed queries regarding lumbar spinal stenosis diagnosis and treatment options [39]. Daher et al. highlighted ChatGPT’s diagnostic potential, indicating reasonable accuracy in identifying shoulder and elbow pathologies. However, they noted a higher accuracy for diagnosis compared to management [28]. In diagnosing cardiovascular conditions, Rizwan and Sadiq observed that ChatGPT showed reasonable accuracy, thereby displaying some clinical utility [41]. Chen et al. illustrated ChatGPT’s capability to offer precise diagnoses and differentials in infectious disease [25], while Vaira et al. demonstrated its accuracy in head and neck surgery contexts [46]. Xv et al. reported that ChatGPT can be used as a tool for diagnosis of common urinary conditions, but included the caveat that it cannot replace residents [48]. A study by Ravipati et al. demonstrated ChatGPT’s proficiency in generating differential diagnoses. However, they noted the model’s diagnostic accuracy for dermatologic conditions was suboptimal [40]. Although LLMs like ChatGPT still have room for improvement, they show promise for diagnostic support. This assistance in the diagnostic process can not only improve efficiency but can also decrease the need for unnecessary tests and ineffective treatments [45].

In addition to diagnostic support, LLMs can augment a physician’s knowledge. This support can come through the summarization of recent literature and clinical guidelines, ultimately providing evidence-based recommendations [33]. Moreover, LLMs have the capacity to deliver complex or specialized information to a provider who lacks expertise in a particular topic, specialty, or pathology, thereby having the potential to act as an initial resource for primary care providers, emergency physicians, or other physicians clinicians faced with unfamiliarity [28,41,49]. LLMs can offer these providers specific information about their patients’ conditions [28], guide further workup [41], and make recommendations for additional specialties to consult [24]. While not a clinical substitute, ChatGPT has the potential to streamline the initial evaluation process, particularly in busy healthcare settings [45,49]. For example, Gebrael et al. discussed ChatGPT’s promising ability to triage patients with metastatic prostate cancer in an emergency room setting [32]. LLMs can help identify red flags in a patient’s presentation that would necessitate immediate medical intervention [31]. This assistance can help ensure prompt attention to high-acuity cases and support informed decision-making [47]. However, further development and refinement are required before LLMs like ChatGPT can be trusted for patient triage [37].

Clinical decision-making regarding patient care and management can also be supported by LLMs. By serving as a quick gateway to the latest research papers [6], treatment guidelines, and in-depth drug information [8,12,35], LLMs can provide physicians with rapid access to relevant information. This can spare physicians from sorting through irrelevant documents or lengthy medical texts and expedite action. Moreover, LLMs can help bridge knowledge gaps, facilitating comprehensive management approaches. In a study by Qu et al., ChatGPT was able to provide a relevant differential diagnosis and reasonable treatment options for otolaryngological conditions [38]. In the ongoing management of chronic conditions, ChatGPT can provide physicians with the means to stay updated on evolving treatment options, thereby potentially enhancing the long-term health outcomes of their patients [35]. Additionally, LLMs can help providers differentiate between different options, such as determining if patients can be managed in an inpatient or outpatient setting [32] and choosing the best immunohistochemistry stain [42]. Furthermore, LLMs have the potential for integration into the healthcare system, enabling continuous patient monitoring [29,33,35]. They can effectively notify both patients and healthcare providers of warning signs indicating possible decompensation or complications and encourage earlier intervention [12,29,35,49]. Additionally, LLMs like ChatGPT can make patient-specific recommendations and develop personalized management strategies [33], ultimately supporting patient-centered care. However, the concern of missing or inaccurate references, sometimes even when providing accurate responses, has been cited [26,36,42,46]. This will need to be addressed with further LLM development to improve transparency.

Outside of direct patient care, LLMs can offer physicians significant support in managing administrative tasks [6,22,23]. Gala and Makaryus note that LLMs can automate note writing and data entry, thereby improving medical record accuracy and minimizing errors. Furthermore, this gives physicians more time to spend with their patients [31]. Streamlining the documentation process can reduce the workload burden for providers and may mitigate burnout [47]. In addition to documentation, LLMs can assist in appointment scheduling [22] and operate as reminder systems [35]. LLMs can also draft routine administrative correspondence, such as referral letters and prescription renewals [35]. Additionally, LLMs may be able to facilitate communication with insurance companies, particularly for preauthorization requests [6]. By improving workflow efficiency and reducing the administrative burden, LLMs will likely enable clinicians to focus more attention on their patients.

### 4.2. Applications of LLMs in Surgical Settings

**Figure 4:**
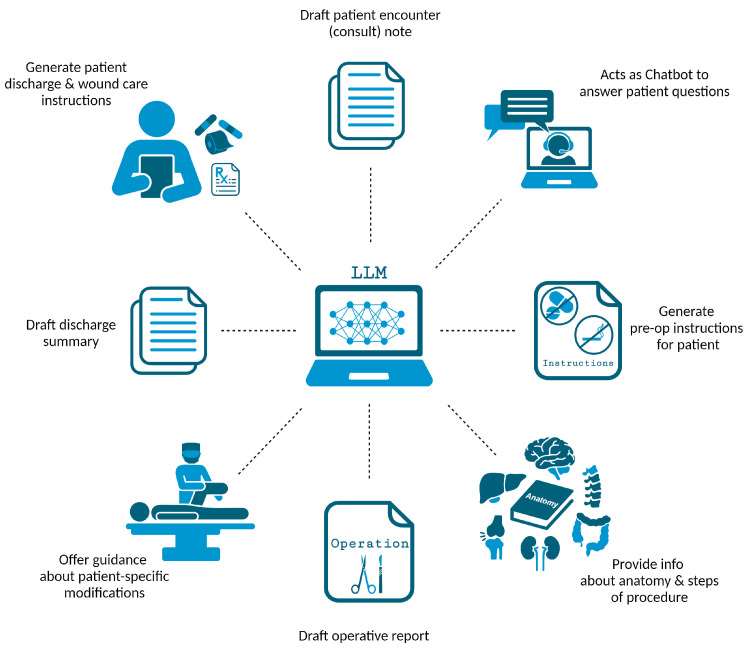
Applications of LLMs within surgical practice. Created with BioRender.com.

LLMs offer a wide array of applications that hold significant relevance for surgeons (Figure 4). Beyond handling routine documentation tasks like composing patient encounter notes and discharge summaries, LLMs can also support surgeons by writing comprehensive operative reports and progress notes [6,31,43]. Additionally, LLMs can help generate perioperative materials [47] like preoperative [35] and postoperative [43] instructions. Since poor discharge summaries and instructions are associated with a higher risk of readmissions and adverse events [43,50], the improvement of written materials using LLMs can have great value. Additionally, LLMs can facilitate communication between patient and surgeon during the informed consent process [6,43] and answer a patient’s surgery-specific questions [35]. LLMs can further support clinical decision-making by guiding the choice between surgical and non-surgical intervention [37,38,39] while also assessing preoperative risk to ensure ideal surgical candidacy [6].

Additionally, LLMs can streamline the surgical planning process and offer real-time notifications to surgeons about crucial perioperative tests, ensuring the best possible surgical outcomes [23,46]. During the perioperative period, LLMs can be used to augment the surgeon’s anatomical knowledge and review critical steps of the surgery, reducing the risk of intraoperative injury [23,51]. Similarly, surgeons can enlist the help of LLMs for strategies to modify a procedure based on patient-specific characteristics [23,46,51]. LLMs can also offer perioperative guidance, such as recommendations regarding thromboembolic prophylaxis [30]. As the list of surgical applications continues to grow, so will the benefits to workflows and surgical outcomes.

### 4.3. Additional LLM Applications in Recent Research

With the rapidly growing volume of literature, numerous relevant studies have been published since the database search of this systematic review. Although most studies echo the same LLM uses, a few new clinical applications have been introduced. In one study, ChatGPT-4 was provided with wrist radiographs and asked to determine whether a distal radius fracture was present, revealing that ChatGPT-4 had a lower sensitivity compared to a hand surgery resident, but a higher precision compared to a medical student [52]. Nonetheless, this study highlights the possibility of using LLMs to assist in medical imaging interpretation. Additionally, support in the classification of hand injuries using ChatGPT-4 and Google’s Gemini has also been explored [53]. Another study investigated ChatGPT’s ability to support surgical planning by predicting the correction angle for medial opening wedge high tibial osteotomy, but the authors noted the model’s performance is currently inadequate [54]. The categorization of surgical patients is another potential application for ChatGPT in the preoperative assessment process [55]. For the innovative surgeon, LLMs like ChatGPT may also be able to assist in the development of patents [56]. Ultimately, these articles highlight the constantly growing list of LLMs applications. However, a consensus remains that despite the considerable potential shown by these models in clinical and surgical applications, their reliable use depends on substantial efforts to improve performance.

### 4.4. Non-Clinical Applications of LLMs in Healthcare

Although not the main focus of this review, it is worth noting that non-clinical uses of LLMs were commonly discussed in addition to clinical ones. Medical education is a domain in which LLMs can offer a wide variety of benefits. They can enable the creation of interactive educational tools, integrating into a medical student’s learning journey [22,35]. LLMs can teach medical students how to draft medical records, and can help non-English-speaking medical students improve their comprehension and writing abilities [12]. Moreover, they can simulate patient cases [7] and facilitate group discussions [12], contributing to an enriched learning experience. LLMs can also help students learn complex concepts and provide personalized instruction and feedback [35,43]. Beyond these applications, LLMs show the potential to enhance medical education by fostering improved communication and problem-solving in clinical settings. Training companies can utilize LLMs to generate new instructional materials and refine existing content, thereby elevating the overall quality of medical education resources [35]. In addition to supporting medical students, LLMs can act as virtual assistants to support resident education [43].

LLMs can also serve as a powerful resource for patients, offering valuable assistance in multiple ways, such as addressing inquiries about medical conditions, providing insights on symptoms and treatment options, and evaluating symptoms to offer guidance on when to seek medical attention [35]. Additionally, LLMs extend their support by assisting patients in the scheduling of medical appointments [22]. LLMs can assume the role of virtual healthcare assistants [22] and may one day be integrated with online health portals [47]. Xiao et al. discuss that as virtual assistants, LLMs can help patients access and understand their health records by answering questions about test results and diagnoses [47]. By improving health literacy and supporting patient autonomy, these LLM applications promise to enhance patient outcomes [7,33,47,49].

LLMs also show remarkable potential in medical research. These models may prove instrumental for researchers, assisting in literature searches and the formulation of innovative research queries [7]. They can streamline literature retrieval and data extraction, simplifying access to relevant information while also condensing lengthy texts into succinct summaries [12,24]. Furthermore, LLMs can expedite the research process by quickly addressing a researcher’s question [41]. Their potential ability to analyze patient data objectively may also facilitate the identification of clinical trial-eligible individuals [29], minimizing selection bias. However, this assumes that the models are purely impartial and do not contain any biases in their training data or algorithms. With further development, LLMs may be able to accelerate the discovery of novel treatments and drug targets while also expediting the development of clinical practice guidelines [22,35]. Additionally, their potential value extends further to support the time-consuming, but important scientific writing process [29,41,47]. LLMs can offer services that include the creation of outlines, proofreading, and critique [26,38]. However, researchers must use LLMs like ChatGPT with caution and verify all outputs. LLMs often struggle to effectively communicate complex and nuanced scientific concepts [12], and responses may be incorrect, outdated, or even plagiarized [7]. Furthermore, LLMs like ChatGPT cannot receive authorship [7], but their involvement in a research project must be diligently acknowledged.

### 4.5. Limitations of LLMs in Healthcare

Although there are many proposed applications and benefits of LLMs, there remains uncertainty regarding their implementation and effectiveness [57]. The reviewed articles collectively emphasize the numerous limitations and concerns associated with the use of LLMs like ChatGPT in healthcare. There was a consensus that accuracy is a major concern, as these models may produce responses that are inaccurate, outdated, or entirely fictionalized, a phenomenon known as “artificial hallucination” [25,32,34,38,39]. Although LLMs like ChatGPT have potential in the guidance of diagnosis and management, there is still much room for improvement. After all, most LLMs like ChatGPT were not originally designed for medical use [37]. Efforts to develop LLMs specifically for medical use is a critical next step. This will require additional investigation to confirm clinical benefit and safety. Such development and validation is required before these models can be approved as medical devices [58].

Various studies have indicated that despite the potential of these models, their current capabilities fall short of the reliability required for dependable use. For example, a study by Chiesa-Estomba et al. reported that ChatGPT could provide accurate responses in the context of their salivary gland clinic, but it would also provide futile treatment recommendations [27]. O’Hern et al. reported that ChatGPT underperformed in the context of triaging dermatologic lesions for Mohs surgery [37]. Furthermore, the lack of standardization in LLM responses can lead to generic, nonspecific, and ambiguous outputs. In their article, Haemmerli et al. noted that ChatGPT displayed potential as a tumor board tool but faced challenges in considering patient-specific nuances [34]. This is consistent with other studies, including an article highlighting that LLMs may offer initial diagnoses for low-risk diseases but face challenges like ambiguity [59]. LLMs may also struggle with contextualizing information and understanding how various pieces of medical knowledge fit together, effectively limiting their ability to address complex conditions, rare disorders, and common illnesses with atypical presentations [35,40].

In addition to inaccurate information, another major concern associated with LLM use in healthcare is the potential to provide and propagate biased information [7,32,35]. These models, trained on extensive but likely biased datasets [3], can mirror societal prejudices in their outputs. This issue poses a risk of influencing clinical decisions and patient care, disproportionately impacting underrepresented groups through biased diagnostic or treatment recommendations [8]. Such biases in LLM outputs could perpetuate existing inequities in healthcare access, quality, and outcomes [22,38,44]. These disparities can also be worsened by the presence of accessibility barriers to LLM use, cost, limited internet access, and language restrictions [29]. Addressing this challenge requires diversifying training datasets, employing bias-detection mechanisms, and integrating ethical guidelines to prevent the widening of healthcare disparities [60]. Additionally, with proper implementation, LLMs may actually be used to minimize healthcare disparities and promote equity [22].

In addition to inequity considerations, there are concerns about LLMs potentially displacing human doctors. Javaid et al. note that with their ability to automate tasks, LLM implementation may result in the elimination of some jobs [35]. However, given their numerous limitations, it is safe to assume that LLMs will not be replacing physicians in the foreseeable future, if they ever do [6,7,35]. Nevertheless, it is important to acknowledge that some patients may turn to ChatGPT for self-diagnosis and self-treatment [31]. Although LLMs like ChatGPT can act as a virtual assistant for patients [22,35], it is unlikely patients will be able to discern inaccurate and potentially harmful information. Therefore, clinician oversight [8] is required to prevent the dissemination of potentially erroneous and harmful information to patients.

While LLMs have made remarkable strides in generating human-like text, it is imperative to recognize their inherent limitations when it comes to replicating genuine human conversation. Despite their capacity to mimic human language patterns, there are concerns that LLMs may fall short in replicating the nuanced complexities of human interaction, particularly in conveying empathy and emotional understanding [22,28,31]. Patients often seek not only information but also reassurance, empathy, and personalized care in their interactions with healthcare professionals. Some literature argues that LLMs can struggle with interpersonal communication [6], lacking human touch and empathy, which can compromise patient trust in both the technology and healthcare providers [22]. In contrast, one study challenging this notion found that ChatGPT was able to provide empathetic responses, even more so than physicians [61]. However, what makes a response empathetic is also likely affected by perception. Therefore, further exploration into this topic is needed for a consensus.

Patient privacy is another significant concern [3,12,22,35,42,43,47]. Upholding patient confidentiality requires strict compliance with regulations such as the Health Insurance Portability and Accountability Act (HIPAA) and rigorous efforts to safeguard patient health information [60]. When information is provided to an LLM, who has access to this information? What safeguarding measures exist? Publicly available LLMs like ChatGPT and Gemini are not currently HIPAA-compliant. While these publicly available models can still offer benefits, the lack of HIPAA-compliance is a major limitation that can compromise the quality of recommendations they provide. Therefore, efforts to develop HIPAA-compliant LLMs should be prioritized [32].

### 4.6. Limitations of This Systematic Review

This systematic review only covers studies that were published as of 14 September 2023. This review does not include the most up-to-date studies due to the continuous output of contemporary research. Although a living systematic review structure would address this limitation, the methodological challenges were deemed to outweigh the potential benefits, and a traditional systematic review was conducted. Nevertheless, proactive efforts were made to expedite the prompt submission for publication to address this limitation. Additionally, we have reviewed the most recent literature and included a few additional studies for discussion.

The review acknowledges the bias favoring positive results in published works, potentially excluding studies where LLMs underperformed. Despite this, the included studies collectively advocated for further development. We also note that although we searched six databases, it is possible that high-quality and pertinent studies existing beyond these sources might have been omitted from this review. Additionally, we imposed the restriction of English-language articles. However, it is important to note that non-English articles accounted for only 0.6% of the initially identified studies. Our eligibility criteria primarily targeted studies primarily examining the utility of LLMs within clinical and surgical contexts. However, it is plausible to acknowledge the possibility of excluding studies that primarily focused on non-clinical or non-surgical aspects but also provided valuable insights into clinical and surgical applications. Furthermore, most included articles were focused on ChatGPT, and there is a paucity of literature on the applications of other LLMs. Therefore, additional investigation into the applications, implementation, and limitations of LLMs is necessary.

### 4.7. Future Directions and Recommendations

The integration of large language models (LLMs) like ChatGPT into healthcare has shown substantial promise across clinical, surgical, and non-clinical domains. Despite the evident benefits and the broad scope of applications highlighted in this review, several challenges and limitations necessitate a directed approach toward future steps and research. The path forward should focus on addressing these challenges while capitalizing on the strengths of LLMs to further enhance patient care, medical education, and healthcare administrative efficiency.

1. Enhancing Accuracy and Reducing Biases. Future research must prioritize the enhancement of LLM accuracy, particularly in clinical diagnosis and management recommendations. Efforts should be directed towards minimizing the occurrence of artificial “hallucinations” and ensuring that the information provided is current, accurate, and evidence-based. Additionally, addressing biases in training datasets is crucial to prevent the perpetuation of discriminatory practices and to ensure equitable healthcare outcomes. This involves diversifying data sources, implementing debiasing methods, and continuously monitoring for bias.
2. Expanding Clinical and Surgical Applications. There is a need for further exploration into the potential applications of LLMs within more specialized medical fields and complex clinical scenarios. Future studies should investigate the integration of LLMs in managing rare diseases and complex cases, as well as providing support in high-stakes surgical planning and decision-making. Research should also explore the feasibility and impact of LLMs in supporting emergency care settings, where rapid and accurate decision-making is critical.
3. Integrating LLMs with Healthcare Systems. Future steps should include the development of interoperable systems that seamlessly integrate LLMs with existing electronic health records. Additionally, future steps should focus on developing secure, HIPAA-compliant, and user-friendly interfaces.
4. Addressing Ethical Concerns. Efforts to resolve ethical concerns related to patient confidentiality, informed consent, and the potential for misinformation are necessary. These ethical concerns should be considered when guiding LLM development and deployment.

## 5. Conclusions

The utilization of LLMs in clinical practice holds promise in optimizing workflow efficiency for physicians and improving healthcare delivery. Although LLMs cannot replace the expertise and clinical judgment of a trained physician, they have the potential to facilitate evidence-based decision-making and enhance the overall quality of patient care. In clinical settings, LLMs have a multitude of different applications in diagnosis, treatment guidance, patient triage, physician knowledge augmentation, and administrative tasks. For surgical applications, LLMs can assist with documentation, surgical planning, and intraoperative guidance. However, there are multiple concerns and limitations surrounding the use of these models, such as the potential for inaccuracy, bias, and violation of patient privacy. Addressing these limitations and ethical concerns is necessary for the responsible use of LLMs. With further development and validation, LLMs and other AI models will be able to serve as valuable healthcare tools.

## Figures and Tables

**Table 1:** Summary of included studies.

First Author	Specialty	Study Design	Objective/Purpose	Main Clinical Application(s) of LLMs	Main Limitation(s) of LLMs	Conclusions
Abi-Rafeh et al. [6]	Plastic surgery	Systematic review of 175 articles	To demonstrate the current and potential clinical uses of ChatGPT in plastic surgery.	Augment clinician knowledge by providing evidence-based recommendations. Assist in clinical note writing, patient triage, interpreting imaging and lab findings, informed consent, and preoperative risk assessment.	Accuracy, reliability, and completeness of provided information. Lack of transparency. Difficulty with interpersonal communication.	Thorough research of proposed applications and limitations of ChatGPT is needed before widespread use in plastic surgery.
Ali [6]	Ophthalmic plastic surgery	Evaluative study	To assess ChatGPT performance regarding lacrimal drainage disorders.	Provide evidence-based information regarding lacrimal drainage disorders.	Content quality relies on training data, impacting accuracy and reliability. Responses may be outdated, verbose, and generic, with potential for discriminatory content. Absence of accountability.	ChatGPT shows average performance in addressing lacrimal drainage disorders yet holds significant potential, necessitating additional development.
Asch [22]	Nonspecific	Interview	To explore ChatGPT’s applications, limitations, and potential impact in healthcare.	Operate as virtual assistants that can answer patient questions, schedule appointments, and provide remote consultations. Expedite the diagnostic process and personalize healthcare. Automate clinical documentation such as medical charts and progress notes.	Data privacy, security, and lack of regulation. Biased responses due to biased data. Interpretability and lack of transparency. Lack of human interaction.	ChatGPT has the potential to improve healthcare delivery, but careful consideration of its challenges and concerns is required before its implementation.
Atkinson [22]	Nonspecific	Systematic review of 34 articles	To explore how Generative AI like ChatGPT can improve medical practice and education.	Improve patient consultations and provide personalized patient care. Streamline physician workflow. Assist in decision-making relating to diagnosis and treatment.	Not adequately discussed.	ChatGPT has potential in healthcare by assisting in diagnosis and management.
Bugaj et al. [23]	Nonspecific	Systematic review of 32 articles	To offer insights into the effect of generative AI-based diagnostic algorithms on patient care based on recent literature.	Assist in clinical decision-making to improve patient care.	Not adequately discussed.	Generative AI, such as ChatGPT, can assess patients and contribute to medical decision-making, resulting in enhanced patient care.
Cadamuro et al. [24]	Laboratory medicine	Evaluative study	To assess ChatGPT’s ability to interpret laboratory results.	Interpret laboratory test results and offer insights regarding deviations. Determine the need for further examination and physician consultation.	Misleading, superficial, and indefinite interpretations. Reluctance to make follow-up recommendations.	ChatGPT can analyze laboratory reports test by test but currently falls short in contextual diagnostic interpretation.
Chen et al. [25]	Neurosurgery	Evaluative study	To evaluate ChatGPT’s ability to assess stroke patients using neurologic scoring systems.	Use established neurologic assessment scales to perform neurologic evaluations.	Accuracy and “hallucinations”. Struggles with complex scenarios.	ChatGPT has potential to assist neurologic evaluations by using established assessment scales. However, occasional inaccurate or “hallucinated” responses currently render it inappropriate for clinical use.
Cheng et al. [26]	Infectious disease	Letter to the Editor	To assess the application of ChatGPT in clinical practice and research within the context of infectious disease.	Disseminate up-to-date information and assist in diagnosis, treatment, and risk assessment. Support telemedicine. Aid in infectious disease surveillance.	Inaccurate or vague answers without references. Regulations.	While ChatGPT holds promise as a tool for clinicians in infectious disease, further development is essential for effective use.
Chiesa-Estomba et al. [27]	Otolaryngology	Prospective cross-sectional study	To evaluate ChatGPT’s capacity to improve management of salivary gland disorders and patient education.	Clinical decision support regarding treatment.	Can produce inaccurate or biased responses. Lack of direct healthcare professional interaction.	ChatGPT shows promise in aiding clinical decision-making and patient information in the salivary gland clinic but needs further development for reliability.
Daher et al. [28]	Orthopedic surgery	Evaluative study	To explore ChatGPT’s potential to diagnose and manage shoulder and elbow complaints.	Diagnosis and management of patients with shoulder and elbow complaints. First consultation resource for primary physicians.	Inaccurate responses. Dependence on imaging results. Lack of up-to-date information.	With its limitations, ChatGPT currently cannot replace a shoulder and elbow specialist in diagnosing and treating patients.
Dave et al. [29]	Nonspecific	Mini review	To explore the practical applications, limitations, and ethical implications of ChatGPT use in healthcare.	Augment a healthcare professional’s knowledge. Assist in generating notes to streamline medical recordkeeping. Assist in diagnosis and clinical decision support.	Can produce inaccurate or biased responses. Potential copyright infringement and other medico-legal issues.	ChatGPT has valuable healthcare applications, but addressing limitations and ethical concerns is essential for effective implementation.
Duey et al. [30]	Orthopedic surgery	Comparative study	To evaluate ChatGPT’s recommendations compared to NASS clinical guidelines regarding thromboembolic prophylaxis for spine surgery.	Perioperative management, specifically thromboembolic prophylaxis recommendations for spine surgery.	Provide recommendations that are incomplete or overly definitive.	ChatGPT shows reasonable alignment with NASS guidelines but requires further refinement for clinical reliability.
Gala and Makaryus [31]	Cardiology	Review	To explore potential applications of LLMs like ChatGPT-4 in cardiology.	Assist in diagnosis and medical decision-making. Facilitate administrative tasks such as documentation.	Provide outdated responses. Lack of contextual understanding. Potential to increase healthcare costs. Lack of accessibility. Lack of human touch and empathy.	ChatGPT has the potential to improve patient outcomes in cardiology. However, limitations and ethical concerns must be addressed for safe use.
Gebrael et al. [32]	Emergency medicine	Retrospective analysis	To evaluate ChatGPT-4’s ability to triage patients with metastatic prostate cancer in the ER.	Analyze patient information to assist in decision-making.	Can produce biased or “hallucinated” responses. Poor disease severity predicting ability. Regulations like HIPAA.	ChatGPT holds promise in enhancing decision-making, such as ER triage, and improving patient care efficiency, but needs refinement for reliable clinical use.
Grupac et al. [33]	Nonspecific	Systematic review of 40 articles	To explore applications of generative AI-based diagnostic algorithms in disease risk detection, personalized healthcare, and patient care.	Augment a clinician’s knowledge by summarizing the literature and clinical guidelines to offer evidence-based recommendations. Assist in clinical decision support regarding diagnosis and treatment. Aid in patient monitoring.	Not adequately discussed.	ChatGPT has potential to provide accurate medical information, supporting clinical decisions, but further exploration is required to assess its limitations and enhance its reliability.
Haemmerli et al. [34]	Neuro-oncology	Evaluative study	To evaluate ChatGPT’s decision-making performance regarding adjuvant therapy for brain glioma.	Provide recommendations for treatment options.	Provide inaccurate, hallucinated, or outdated responses. Can make ineffective or harmful recommendations. Can struggle to accurately identify glioma subtype and consider functional status.	ChatGPT has potential as a supplemental tool by providing valuable adjuvant treatment recommendations, but has limitations.
Javaid et al. [35]	Nonspecific	Literature review	To explore applications of ChatGPT within healthcare.	Can access patient information to provide medical suggestions and counseling. Develop patient-specific treatment programs. Offer medication reminders and assist in remote patient monitoring. Schedule appointments.	Can produce inaccurate or biased responses, thereby spreading misinformation. Ethical and privacy concerns. Can struggle with complex or abstract scenarios.	ChatGPT shows promise in various healthcare applications. However, addressing limitations is crucial for maximizing its potential.
Kottlors et al. [36]	Radiology	Evaluative study	To evaluate GPT-4’s ability to generate differential diagnoses based on imaging patterns.	Generate differential diagnoses based on medical imaging.	Lack of transparency. Verification of references.	LLMs like ChatGPT-4 can provide differential diagnoses based on imaging patterns, ultimately showing promise in diagnostic decision-making.
Muftić et al. [3]	Nonspecific	Systematic review of 31 articles	To explore ChatGPT’s ability to streamline tasks, optimize clinical decision-making, and facilitate communication, ultimately improving patient care.	Facilitate inter-professional communication. Assist in clinical decision-making to improve patient care.	Can produce inaccurate or biased responses. Can struggle with prompts that are lengthy, image-based, in a different language, or contain medical terminology. Patient privacy.	ChatGPT holds promise in diverse medical applications, but addressing challenges and limitations is essential for safe implementation in healthcare.
O’Hern et al. [37]	Dermatology	Letter to the Editor	To assess ChatGPT’s ability to effectively triage surgical management for patients with cutaneous neoplasms.	Triage patients with cutaneous neoplasms and guide treatment.	ChatGPT was not designed for medical use. Limited congruency with established guidelines (e.g., MS AUC).	ChatGPT demonstrates limited proficiency in triaging surgical options for cutaneous neoplasms, highlighting the importance of cautious application in clinical decision-making.
Qu et al. [38]	Otolaryngology	Cross-sectional survey	To assess ChatGPT’s clinical applications and limitations within otolaryngology.	Support diagnosis and management in otolaryngology.	Responses may be inaccurate, “hallucinated”, biased, or outdated.	ChatGPT can provide differential diagnoses and treatment options in otolaryngology, however limitations must be addressed.
Rajjoub et al. [39]	Spine surgery	Comparative analysis and narrative review	To evaluate ChatGPT’s recommendations compared to NASS clinical guidelines regarding diagnosis and treatment of degenerative LSS.	Assist in decision-making relating to diagnosis and treatment.	Responses may be inaccurate, “hallucinated”, biased, or nonspecific.	ChatGPT shows potential in assisting clinical decision-making for LSS diagnosis and treatment, but requires further standardization and validation.
Ravipati et al. [40]	Dermatology	Letter to the Editor	To evaluate ChatGPT’s accuracy and reliability in diagnosing dermatologic conditions.	Assist in diagnostic support for dermatologic conditions, such as generating differential diagnoses.	Responses may be inaccurate. Can struggle with prompts that are image-based.	ChatGPT demonstrates potential as a differential diagnosis generator, but requires refinement before its application in dermatology.
Rizwan and Sadiq [41]	Cardiology	Evaluative study	To investigate ChatGPT’s potential to assist providers with diagnosis and treatment of cardiovascular disorders.	Assist in decision-making relating to diagnosis and treatment of cardiovascular disease.	Not personalized, as responses can be nonspecific and incomplete.	ChatGPT can provide comprehensive, understandable responses with academic and clinical benefits, yet its limitations require attention.
Sallam [7]	Nonspecific	Systematic review of 60 articles	To examine ChatGPT’s utility and limitations within healthcare, research, and medical education.	Assist in decision-making relating to diagnosis and treatment.	Ethical, copyright, and transparency issues. Risks of bias, plagiarism, and inaccurate content.	ChatGPT has potential to streamline healthcare, but its adoption requires caution due to limitations and ethical considerations.
Schukow et al. [42]	Diagnostic pathology	Literature review	To explore ChatGPT’s potential advantages and disadvantages in diagnostic pathology.	Summarize diagnostic queries and enhance subspecialty inquiries. Assist in decision-making relating to diagnosis and treatment.	Responses may be incorrect and lack references. Patient privacy.	ChatGPT shows promise in diagnostic pathology, but its reliability and ethical use must be carefully considered.
Sharma et al. [43]	Plastic surgery	Literature review	To assess ChatGPT’s utility within plastic surgery.	Assist with clinical tasks and healthcare communication.	Responses may be incorrect or outdated, leading to misinformation. Plagiarism. Patient privacy.	ChatGPT can improve productivity in plastic surgery, but requires further development and cautious implementation.
Sorin et al. [44]	Oncology	Retrospective study	To assess ChatGPT’s role as a decision-making support tool for breast tumor boards.	Clinical decision support in breast tumor board meetings. Assist in summarizing patient cases and providing management recommendations.	Inconsistent recommendations. Biased responses due to biased data	ChatGPT has potential as a decision support tool, aligning with tumor board decisions, but further validation is required.
Srivastav et al. [45]	Radiology	Systematic review of 39 articles	To offer an overview of AI, particularly ChatGPT, in radiology and medical imaging diagnosis.	Enhance diagnostic accuracy and minimize errors to improve workflow efficiency.	Data quality and ethical concerns.	ChatGPT has potential to improve radiological diagnoses and patient care, but requires further research and development.
Tustumi et al. [8]	Gastrointestinal	Narrative review	To explore ChatGPT’s applications in disease diagnosis, treatment, prevention, and the development of clinical practice guidelines.	Can augment diagnostics and patient management. Can accelerate the creation of clinical practice guidelines.	Biased responses due to biased data. Need for human oversight.	While ChatGPT shows promise in healthcare, oversight and awareness of limitations is needed. Additionally, the model cannot replace healthcare professionals.
Vaira et al. [46]	Head and neck surgery	Observational and evaluative study	To evaluate ChatGPT’s accuracy in addressing head and neck surgery questions and clinical scenarios.	Assist in decision-making relating to diagnosis and treatment planning for head and neck surgery. Support patient counseling.	Inconsistent or incomplete recommendations. Lack of references.	ChatGPT shows promise in head and neck surgery but needs more development and validation to be a reliable decision aid.
Xiao et al. [47]	Pediatric surgery	Literature review	To explore ChatGPT’s potential in pediatric surgery research and practice.	Assist in decision-making relating to diagnosis and patient care. Facilitate administrative tasks such as documentation.	Responses may be inaccurate, unreliable, or outdated. Patient privacy.	ChatGPT offers potential in healthcare and pediatric surgery for efficiency and support, yet demands further development for effective integration.
Xv et al. [48]	Urology	Letter to the Editor	To evaluate ChatGPT’s ability to diagnose urinary diseases compared to urology residents.	Assist in the diagnosis of urinary system diseases.	Not adequately discussed.	ChatGPT can act as a supplementary tool for diagnosing common urinary diseases, supporting rather than substituting healthcare professionals.
Zhang et al. [12]	Gastrointestinal	Review	To explore ChatGPT’s applications and limitations in healthcare.	Enhance diagnostic accuracy and efficiency. Assist in treatment and patient care. Support public health initiatives.	Responses may be inaccurate or outdated, leading to misinformation. Patient privacy.	ChatGPT demonstrates potential in healthcare through professional support with reliable information, yet addressing its limitations is essential for widespread clinical use.

Abbreviations: ER, emergency room; HIPAA, Health Insurance Portability and Accountability Act; LLMs, large language models; LSS, lumbar spinal stenosis; MS AUC, Mohs surgery appropriate use criteria; NASS, North American Spine Society.

## Data Availability

The data that support the findings of this study are available from the corresponding author upon reasonable request.

## Abbreviations

AI	artificial intelligence
CINAHL	Cumulative Index to Nursing and Allied Health Literature
EMBASE	Excerpta Medica Database
GCS	Glasgow Coma Scale
GPT	generative pre-trained transformer
HIPAA	Health Insurance Portability and Accountability Act
LLM	large language model
LSS	lumbar spinal stenosis
MS AUC	Mohs surgery appropriate use criteria
NASS	North American Spine Society
NLP	natural language processing
PRISMA	Preferred Reporting Items for Systematic Reviews and Meta-Analyses
USMLE	United States Medical Licensing Examination
